# From the concrete to the intangible: understanding the diverse experiences and impacts of new transport infrastructure

**DOI:** 10.1186/s12966-015-0230-4

**Published:** 2015-06-04

**Authors:** Joanna May Kesten, Cornelia Guell, Simon Cohn, David Ogilvie

**Affiliations:** MRC Epidemiology Unit and UKCRC Centre for Diet and Activity Research (CEDAR), University of Cambridge School of Clinical Medicine, Box, 285, Cambridge Biomedical Campus, Cambridge, CB2 0QQ UK; Department of Health Services Research and Policy, London School of Hygiene and Tropical Medicine, 15-17 Tavistock Place, London, WC1H 9SH UK

**Keywords:** Active travel, Behaviour change, Commuting, Environment, Evaluation, Natural experimental study, Public transport, Physical activity, Qualitative, Transport

## Abstract

**Background:**

Changes to the environment that support active travel have the potential to increase population physical activity. The Cambridgeshire Guided Busway is an example of such an intervention that provides new traffic-free infrastructure for walking, cycling and public transport. This qualitative investigation explored the diverse experiences of new transport infrastructure and its impacts on active travel behaviours.

**Methods:**

Thirty-eight adult participants from the Commuting and Health in Cambridge natural experimental study were purposively selected according to their demographic and travel behaviour change characteristics and invited to participate in semi-structured interviews between February and June 2013. A mixed-method, following-a-thread approach was used to construct two contrasting vignettes (stories) to which the participants were asked to respond as part of the interviews. Inductive thematic qualitative analysis of the interview data was performed with the aid of QSR NVivo8.

**Results:**

Perceptions of the busway’s attributes were important in shaping responses to it. Some participants rarely considered the new transport infrastructure or described it as unappealing because of its inaccessibility or inconvenient routing. Others located more conveniently for access points experienced the new infrastructure as an attractive travel option. Likewise, the guided buses and adjacent path presented ambiguous spaces which were received in different ways, depending on travel preferences. While new features such as on board internet access or off-road cycling were appreciated, shortcomings such as overcrowded buses or a lack of path lighting were barriers to use. The process of adapting to the environmental change was discussed in terms of planning and trialling new behaviours. The establishment of the busway in commuting patterns appeared to be influenced by whether the anticipated benefits of change were realised.

**Conclusions:**

This study examined the diverse responses to an environmental intervention that may help to explain small or conflicting aggregate effects in quantitative outcome evaluation studies. Place and space features, including accessibility, convenience, pleasantness and safety relative to the alternative options were important for the acceptance of the busway. Our findings show how environmental change supporting active travel and public transport can encourage behaviour change for some people in certain circumstances.

## Background

In recent years there has been growing interest in the opportunity to increase population levels of physical activity by targeting travel behaviours [1,2]. Active travel—in particular for commuting, which accounts for 19 % of all trips made in the United Kingdom (UK) [3]—is associated with higher overall physical activity [4-8] and physical wellbeing [9] and lower cardiovascular disease risk factors [10-12]. Commuting by walking or cycling is associated with a lower risk of being overweight [12], and this includes the use of public transport that can involve walking [13] and cycling [14] as part of the journey, thereby contributing towards the achievement of recommended physical activity levels [15].

### Evaluating the physical activity impacts of environmental changes

Studies of the effects of interventions to promote walking [16] and cycling [17] suggest that these behaviours are amenable to change in principle, and it is increasingly argued that creating a more supportive environment for these behaviours should form a key part of public health strategy in this area [4]. However, more robust studies are needed to assess and understand the effectiveness of population level interventions to promote physical activity [16,18]. Randomised controlled trials are not always possible, practical or appropriate for evaluating large-scale environmental changes [19] and it is often necessary to use alternative study designs. Natural experiments—defined in this case as changes in exposure to environmental conditions that are not manipulated by the researcher [19,20]—can be useful for assessing the effects of environmental interventions on health [19] and health inequalities [20]. The health impacts of natural experiments, addressing changes such as residential relocation [21], housing improvements [22] and new transport infrastructure [23,24], are increasingly being evaluated. The complexity of natural experimental studies means that multiple methods, including quantitative and qualitative approaches, are often recommended [20], and quantitative outcome studies in this area are prone to producing modest, mixed or inconclusive evidence of aggregate behaviour change which may conceal divergent patterns of response between different groups of people exposed to the interventions [25]. Qualitative methods are particularly useful for understanding attitudes to and processes of change, and for their contribution to the interpretation of quantitative analyses [20], but to date their use in evaluating the physical activity impacts of environmental changes has been limited [26,27]. Understanding how change is brought about, experienced and maintained, and under what circumstances, is important for the development of more generalisable causal inference and policies to promote and sustain more widespread population level changes [28-30].

### The case of the Cambridgeshire guided busway

**Fig. 1:**
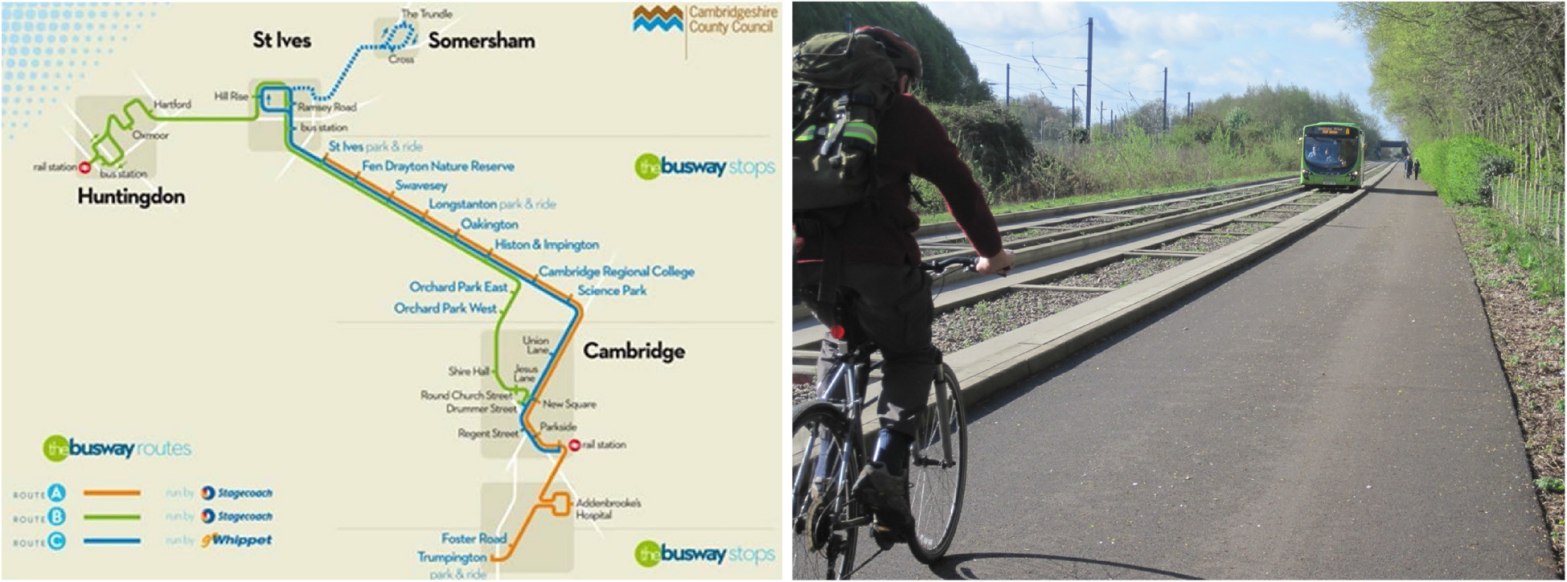
Cambridgeshire Guided Busway route and image. Credit for map: Cambridgeshire County Council. Reproduced with permission

One example of an environmental change designed to promote active travel and public transport is the Cambridgeshire Guided Busway in the UK (henceforth the busway). The relative lack of affordable housing in Cambridge means that it is common for people who work in Cambridge to live in the surrounding towns and villages. The resultant car commuting into Cambridge causes considerable congestion on main roads (such as the A14 and M11) and ‘rat running’ through surrounding villages [31]. Built on two disused rail tracks, the busway is a transport system that links Huntingdon, Cambridge and Trumpington, consisting of a dual-lane dedicated track for buses and a ‘maintenance track’ for pedestrians, cyclists and horse riders (Fig. 1). The buses are adapted with guided bus technology, ensuring uninterrupted contact between the bus and the kerb of the track (a feature which makes the ride smooth), whilst also allowing the use of normal roads through the city centre and beyond the terminus of the busway to the surrounding towns and villages. The busway and maintenance track offer a traffic-free, off-road route linking workplaces to parts of the commuter belt and to park-and-ride facilities.

The Commuting and Health in Cambridge study aims to understand the impact of the busway on travel behaviour, physical activity and wider health. The study protocol has been published elsewhere [32]. Briefly, it is a quasi-experimental cohort study of commuters in which data were collected in four annual surveys augmented with nested in-depth quantitative and qualitative components. Participants aged over 16, travelling to work in Cambridge and not participating in other concurrent physical activity research were recruited, predominantly through workplaces, and invited to participate in annual questionnaire surveys and (optional) rounds of objective physical activity measurement between 2009 and 2012, that is covering the period before and after the busway was opened in 2011. A complementary intercept survey of busway users was also conducted in 2012 to assess who used the busway, for what purposes and how such journeys were made prior to the busway. In addition to the cohort study and intercept survey, qualitative interviews were conducted in each year of the study and have provided insights into the social context of commuting practices [33], the socioeconomic structure of car commuting [34], depictions of wellbeing associated with commuting [35], the resilience of active commuters to apparently hostile commuting environments [36], factors underlying changes in commuting practices following home or work relocation [37] and the initial experiences of busway users [38]. The current paper focuses on the analysis of qualitative interview data collected after the busway was opened, investigating the ways in which people experienced and responded to the new transport infrastructure, and how such experiences were or were not translated into meaningful travel behaviour change.

## Methods

### Setting

Cambridge is a university city in the east of England and has a resident population of 123,867 [39]. It is characterised by a strong cycling culture. A high proportion of commuters (29.9 %) reported cycling to work in the 2011 census compared to 2.8 % in England and Wales as a whole [40], and fewer commuting journeys are made by car in Cambridgeshire (50 %) than in Great Britain as a whole (68 %) [41].

### Interview sampling procedure

Interview participants were recruited from the main study cohort and the intercept survey sample. Those recruited from the cohort were purposively sampled from among those who had taken part in at least two of the annual surveys. They were categorised according to their responses to the survey question ‘*In the past four weeks how did you normally travel to work?*’, to which they could respond ‘*always, usually, occasionally* or *never*’ in respect of each of four modes of transport (*car or motor vehicle, public transport, bicycle* or *walking*). This enabled us to distinguish those who had changed or maintained their usual mode of travel to work over the course of the study. The intercept survey participants were more socioeconomically heterogeneous than the cohort sample and were known to have used the busway, which was not the case for all the members of the cohort. Intercept survey participants were eligible for inclusion in the interview study if they were aged over 16, lived within the study area and had reported their level of educational achievement, which enabled us to purposively oversample from lower social groups. Across the two sources the aim was to recruit a sample representing a broad range of characteristics including age, education, gender, and home location. The latter was used to classify participants according to their level of exposure to the busway into loosely-defined notional ‘intervention’ and ‘control’ areas [29].

### Interview procedure

Semi-structured interviews were conducted between February and June 2013 (between 18 and 22 months after the busway was introduced), either at the participants’ homes (26.3 %) or workplaces (60.5 %) or at the research institute (13.2 %). The interviews followed a flexible topic guide which allowed participants to shape the direction of the interview depending on their responses [42] and lasted between 18 and 71 minutes. Interviews were conducted until theoretical saturation (when little new information was emerging) had been reached [43]. The participants were not offered any form of compensation for their time. The Cambridge Psychology Research Ethics Committee Ethical granted approval for the study (reference number PRE.2012.14) and all participants provided written informed consent to take part in an interview.

The interviews began by exploring participants’ experiences of using different modes of transport and how they chose between the options available to them. The interviews then focused more closely on the facilitators of, barriers to and processes of travel behaviour change, with the more specific question of the perceived impact (if any) of the busway being raised towards the end of the interview if it had not been mentioned spontaneously by the participant.

Vignettes were employed at the end of the interviews. Vignettes are descriptions of events or stories which are used to elicit a participant’s response and reaction [44]; they are similar to observing another’s behaviour, and can encourage participants to identify with the story’s protagonist and consider what they might do or feel in a similar situation [45]. To construct the vignettes the ‘following a thread’ procedure for mixing methods was adopted [46]. The quantitative predictors of the maintenance and uptake of travel behaviours in the cohort study [47] were used to re-analyse and deductively extract existing qualitative data previously collected in the study [33, 35, 37]. This information was then iteratively combined and modified to produce two coherent ‘moving portrait’ vignettes (Table 1), presenting the words of the participants (portrait) with a particular emphasis on the flow of events over time (moving) [48]. The first vignette focused on positive attitudes towards active travel and the use of public transport. A contrasting vignette, depicting the experience of car use, was developed using data relating to the inverse of the quantitative predictors. Producing the vignettes using empirical quantitative and qualitative data in this way was intended to make them as credible, accurate and relevant as possible [44].

**Table 1:** Vignettes used to elicit discussion in the interviews

Vignette 1. Decreasing car use
I mean it’s really in the last five years my attitude’s changed to public transport. There’s a very good bus route, coming into the centre of Cambridge so I either take the bus or cycle in to work, depending on how the weather is. When the weather is not nice, the bus that I take leaves at about five past nine and arrives here at half past nine-ish, depending on the traffic. And I do that so that I can take my son to school for nine o’clock and then take that bus. It was a real change in my behaviour when they introduced these buses. The old buses were a lot less comfortable, they didn’t have any air conditioning, so I often felt sick when I was reading on the bus, these buses have air conditioning, they’re very well kitted out. They’ve got lots of leg space and plug sockets for you to plug your laptop in. And by having access to the internet, I basically start work as soon as I get on the bus. I opt for the bike in the Summer because I’ve always enjoyed cycling. I'm not someone who will exercise for the sake of exercising, I don't enjoy it and don’t tend to stick to it so doing it this way, as the commute to work, it means that I’m doing exercise consistently for a reason and I’ll stick to it.
Vignette 2. Continuation of car use
The last few years I’ve been driving and I love driving, I drive everywhere. Having the car gives you much more freedom, especially with a child. One reason for driving is a lack of ideal public transport. Deciding between driving and other options is like a balance between the convenience of a car which can literally get you from door to door, with trying to do the green thing and taking public transport or cycling. I didn’t always drive everywhere, I used to cycle to work from a park-and-ride, until a few years back when I fell off my bicycle and now my wife won’t let me cycle in town. Comparatively cycling is not actually much different to the car because I’d still leave the same time and I’d probably arrive at the same time because of that last bit coming into Cambridge the traffic is probably comparable. It’s just the slight inconvenience of cycling and having to change when you come to work.

### Analysis

Inductive thematic qualitative analysis was performed using QSR NVivo 8 [49]. This approach was chosen because it offers clear guidance on the process of analysis whilst remaining flexible and encouraging the comparison of connections and divergence within the data [49]. Reflective field notes, recording the main points of interest and unrecorded talk (e.g. before and after audio recording), were completed after every interview. The field notes were referred to for context before, during and after analysing each transcript. Initial codes, categorising the content within each line or section, were generated systematically across all the transcripts, and duplicate codes with synonymous meanings were collapsed. The content of all the codes was read, and these contents were compared to each other to iteratively refine and group codes into potential themes. To continue the refinement process the content of each theme was used to produce a written description of each theme. This description recorded instances of divergent cases and helped to ensure that the content reflected the theme accurately and that the theme was an accurate description of the content. During this process the experiences of one particular participant emerged as a useful illustration (case study) of the positive potential impact of the busway. Participants were given pseudonyms for the purposes of reporting.

## Results

### Sample characteristics

In total 132 participants were invited to participate in an interview (100 intercept survey participants and 32 cohort members) of whom 38 consented (28.8 %). A higher proportion of cohort members (71.9 %) than intercept survey participants (15.0 %) agreed to be interviewed. Twenty-one (55.3 %) of the participants were women; their ages ranged from 30 to over 70, with 17 (42.1 %) in their fifties; and the majority (~80 %) had completed secondary school or higher education (Table 2). Eleven of those recruited from the intercept survey had not been educated beyond secondary school level, and four were unemployed. Based on their interview data, the participants could be classified as having not significantly changed their travel behaviour over time (*n* = 11); having shifted towards more active travel or less car travel (*n* = 13); having shifted towards less active travel or more car travel (*n* = 10); or having shifted in a manner less directly affecting physical activity or car use, for example by shifting from conventional bus to guided bus (*n* = 4) (Table 3). Twenty-six participants (68.4 %) had used the guided bus and/or maintenance track with varying levels of regularity.

**Table 2:** Participant characteristics (*n* = 38)

Characteristic	Description	N (%)
Gender		
	Male	17 (44.7)
	Female	21 (55.3)
Age (years)		
	30–39	7 (18.4)
	40–49	6 (15.8)
	50–59	16 (42.1)
	60–69	7 (18.4)
	70 and over	2 (5.3)
Employment		
	Employed	35 (92.1)
	Unemployed^a^	3 (7.9)
Education		
	Higher education	19 (50.0)
	Secondary education	12 (31.6)
	Other qualification	4 (10.5)
	None	3 (7.9)
Area of residence		
	Notional intervention area	19 (50.0)
	Notional control area	19 (50.0)
Recruitment group		
	Intercept	15 (39.5)
	Cohort	23 (60.5)
Behaviour change		
	None	11 (28.9)
	More active (away from car)	13 (34.2)
	Less active (towards car)	10 (26.3)
	Change which does not affect activity levels	4 (10.5)
Interviewed previously		
	Yes	3 (7.9)
	No	35 (92.1)
Total		38

^a^Includes: retired or looking after home or family

**Table 3:** Profile of participants’ travel behaviours

Participant pseudonyms	Use of guided busway	Current journey description	Change and main reason described
			None (*n* = 11)
Hannah	No	Drove to work	N/A
Lucy	No	Drove to park-and-ride and then walked to work	N/A
Beth	No	Drove to work	N/A
Meg	No	Drove herself and her husband to his workplace where there is free parking and then either walked or cycled to work	N/A
Matt	Bus	Took public transport to work	N/A
Tara	Bus	Drove to work	N/A
Jasmine	Path	Cycled to work	N/A
Jamie	Path	Cycled to work	N/A
Graham	Bus	Usually drove	N/A
Daniel	Bus	Worked from home but travelled to see clients predominantly by car	N/A
Robert	Bus	Cycled to the train station, then took a train and then a bus to work	N/A
			Change which does not affect activity levels (*n* = 4)
Mark	Bus and path	Usually cycled or took the guided bus and drove occasionally	Previously used normal bus system, cycled on the road and drove occasionally. Changed to using the guided bus and maintenance track to cycle to work.
Holly	Bus	Used the guided bus	Changed from using the normal bus to the guided bus when the weather was nice or when her husband drove her to the park-and-ride.
Paula	Path	Either cycled or used a bus	Took up cycling to work partly along the guided busway. Travelled by bus in the winter.
Liam	Path	Drove to work and occasionally cycled along the busway	Took up cycling to work occasionally after introduction of the maintenance track.
			More active (away from car) (*n* = 13)
Oliver	Bus	Either cycled or drove to work	Took up cycling approximately three years ago after his wife started cycling and suggested that he try it too.
Zoe	No	Cycled, drove, or drove to a park-and-ride and then cycled	Change prompted by retirement of partner with whom she travelled by car to work. Changed to cycling, or driving to park-and-ride and then cycling.
Freya	Bus and path	Cycled to work	Changed from driving to park-and-ride and cycling to work to cycling the entire journey — either on the busway or on the normal roads depending on the weather which determined whether the access path to the busway was passable.
Harry	Bus and path	Cycled or took the guided bus to work	Change primarily caused by moving house which made journey longer, thereby increasing cycling distance.
William	No	Either drove or cycled to work	Changed from driving to work all the time to occasionally cycling. Change primarily caused by moving house.
Catherine	No	Drove to work and occasionally cycled to work	Changed from driving to park-and-ride and then taking a bus to work to mainly driving and occasionally cycling. Change caused primarily by moving house which shortened the journey.
Peter	Path	Cycled to work	Changed from taking a train and cycling part of the journey to cycling the entire journey. Change primarily caused by moving house.
Helen	No	Drove to park-and-ride and walked the rest of the way to work	Changed from driving the entire journey to work to driving to a park-and-ride site and walking the rest of the way to work. Change primarily caused by removal of workplace parking.
Vicky	Bus	Drove to park-and-ride and then took a bus to work. Walked the return journey to park-and-ride site	Changed from parking on site to using park-and-ride and taking a bus to work and walking the return journey. Change driven by the stress of trying to find a parking space.
Nick	Bus	Used the guided bus four days a week and drove to a different workplace once a week	Previously car-shared with his wife who would drop him at work and would continue to her own workplace. Changed from driving to work to taking the busway after moving home. This change involved more walking.
Jenny	Bus	Drove to park-and-ride and took the guided bus to work	Previously drove from home to work. Decided to try out a park-and-ride and cycle along a narrow cycle path which ran alongside a road. When the busway was introduced she drove to the park-and-ride and cycled along the busway path which she preferred because it was wider. In the winter she decided to try the guided bus from her home to her workplace.
Omar	Bus	Either drove to work or drove to a park-and-ride and walked or took the guided bus the rest of the way to work	Change brought about by health concerns which led to increased willingness to use the park-and-rides more to walk. Started using the guided busway once he had a free bus pass and reduced the amount of cycling he did to work due to health problems.
Paul	Path	Drove for food shopping	Decreased driving, especially long distances, owing to health problems. Took up cycling for leisure, which he attributed to the Olympics.
			Less active (towards car) (*n* = 10)
Alice	Bus	Drove to work	Change caused by changing jobs three times during the study. When participant had to pay for parking at work she either took the train or drove to a park-and-ride and then walked or cycled to work. Changed to driving every day due to free parking at her work place.
Greg	Bus and path	Took a bus to work	Changed from cycling to work to taking public transport. Change caused by increasing age and health problems meaning he was less able and inclined to cycle to work.
Louise	No	Drove to work	Participant used to take two buses from home to work. Due to health problems she occasionally needed to use car to get to work. Employers granted her a permanent parking permit so she now drives to work.
Hester	No	Drove to work	Used to cycle to work three times a week when the weather was nice and drove when she was going on to appointments. Since semi-retiring she had been cycling more due to decreased work pressures but she had broken her arm a few months ago and hadn't been able to cycle since.
Kevin	No	Either drove to work or took two buses	Primarily cycled to work before moving home. After moving home cycling to work was no longer possible due to increased distance.
Leah	Path	Drove an electric scooter to work, occasionally drove a car or cycled to work	Primarily cycled to work before moving home. After moving home and purchasing an electric scooter and needing to drive more as a result of changes to job, she cycled to work less.
Kayleigh	No	Drove to work	Changed from driving to park-and-ride and cycling the rest of the way to work, to predominantly using the car and occasionally taking the bus to work. Change provoked by bicycle being stolen from the park-and-ride and second bicycle getting a puncture.
Sally	Bus	Drove to work	Participant had changed from taking a train and walking to work to driving. Change attributed to learning to drive.
Sophie	Path	Cycled with children to school	Participant had changed from cycling to work to working from home and therefore not needing to travel to a work place.
George	Bus and path	Drove to work	Change driven by change in job, which meant he could no longer cycle to work because of the increased distance.

The following three main themes were elicited from the interviews: ‘Places created by environmental change’; ‘Ambiguous spaces created by environmental change’; and ‘Adapting to and adopting environmental change’. These substantive themes are described and discussed using illustrative quotes including each participant’s pseudonym and age group. In addition to these substantive themes, the case study of one participant is highlighted to illustrate the potential impact of the busway, and this is followed by a reflection on the contribution of the vignettes to the study.

### Places created by environmental change

#### Location relative to environmental change

The introduction of the busway created places, connections between places and connections between people and places. Some participants did not commonly use the busway, while others had embraced it as a new, more convenient, transport option. For both groups, perceptions of the attributes of the busway—its proximity, accessibility and convenience—were important in shaping their response to it.

Some participants did not use the busway or the maintenance track because they lived far from the route or from feeder modes or interchange facilities such as connecting bus services and park-and-ride sites.

*“The [guided] bus route has no relevance or bearing to me at all, it’s the wrong direction and the wrong place and anything like that so I couldn’t use it even if I wanted to particularly.”* Alice, 50–59 years

Interestingly this included not only participants from the ‘control area’ but also intercept survey participants who were sampled through use of the very infrastructure they claimed not to be using, suggesting that they may have been intercepted on the busway on a rare or non-routine occasion.

In contrast, for other participants the new transport infrastructure was located on their commuting route and was therefore able to replace previous options altogether; and expansions of the busway route network meant that the area within which the busway was accessible increased over time.

*“In 2009 I would have driven to work and parked on site, because that’s really the only option. I live about 23–25 miles away, and the buses would have taken me quite a long time to get to work, so I would have driven and parked on site. Then there was the opportunity to park at the Trumpington Park-and-Ride and cycle along the guided busway, so I opted to try some of those.”* Jenny, 60–69 years

#### Other place-related considerations

People who experienced the guided busway as convenient described appreciating the fact that compared with normal public transport, the busway services were more frequent, made fewer stops and took a more direct route. Similarly, the maintenance track provided a convenient, easy to use, smooth cycle path away from roads, which meant fewer stops at road junctions.

*“If you went to Trumpington and tried that six minute [bus] journey, you’d appreciate how, when it’s not got on the road or there’s nothing in the way, how efficient it is and what it could aspire to.”* Paul, 70–79 years

*“[The maintenance track]’s quite convenient the way it avoids quite a lot of junctions and stopping and starting because you’re coming into town, you peel straight off into the park-and-ride and there’s very little stopping for traffic lights and junctions.”* Peter, 30–39 years

Although the busway was meant to bypass traffic congestion, a proportion of the route followed by the guided buses—through the city centre of Cambridge—was ‘on road’ and had therefore not succeeded in providing an altogether faster or more reliable travel option for many.

*“But most of the time one could get to [my work place], as an example, just as quickly going the traditional car route than if you were to go on the bus [busway]. Because once it [the busway] hits the city in Cambridge, it goes all around the houses […]”* Daniel, 50–59 years

This experience depended very much on a participant’s home or work location and on whether the on-road portion of the busway route formed a significant part of the overall journey.

The introduction of the busway may thus have acted to change the balance of influential factors in travel behaviour choices. The relative importance placed on these factors depended on the individual, their social circumstances and other influential considerations. For some, for example, stress caused by driving to work on congested roads and difficulties parking was reduced by incorporating the busway into their commute.

*“I’ve worked on this site for about 15 years, and over the years it’s beenverystressful getting a car parking place, even if you come in early. And I just can’t start my day in a stressful way, so Park and Ride is really good for me, getting on the [guided] bus is very, very good.”* Vicky, 50–59 years

In summary, this evidence suggests that the new infrastructure cannot be considered as a singular change to the environment that affected everyone’s choices and opportunities in the same way [50]. What was regarded as a novel, quasi-tramway service by some was experienced as no different to an ordinary bus service by others—illustrating that perceptions of the nature of the ‘intervention’ embodied by the busway varied within the study population and resulted in diverse effects influenced by individual circumstances and the value individuals attributed to certain factors.

### Ambiguous spaces created by environmental change

Rather than the *place* of this new infrastructure, it was the *space* that it created which elicited either acceptance or objection from participants.

#### A more pleasant travel option?

Overcrowding on buses and proximity to other people were described as particularly unappealing and acted as a barrier to use, especially when considered alongside the price of tickets. These participants expressed a general dislike of public transport, and saw no notable difference to regular bus services in terms of comfort or pleasantness.

*“The principle of it is terrific and I know from things that I’ve read that the volume of people that are using the busway has increased many fold and that’s fabulous […]. But my experience of public transport generally is that it’s usually not in my favour. […W]ith public transport you end up being in other people’s private space […] and you’d have hot, sweaty people in the summer and cold, sneezing people in the winter.”* Daniel, 50–59 years

Negative attitudes towards public transport were also echoed in these participants’ discussion of the widely advertised features of the new busway, such as free internet access and power sockets. They did not regard these features as positive assets or outweighing their concerns about discomfort, and some did not even know about them.

*“[…] plugging your laptop in [on the bus] and starting to work, I can’t think of anything worse […].”* Catherine 50-59 years

Similar narratives were shared by some participants in terms of the use of the maintenance track, and cycling and walking as modes of transport in general. In particular, those who did not regard cycling as an acceptable mode of transport tended not to consider the maintenance track as an option.

*“[…] it’s not so much I don’t like cycling as the fact that I don’t like wearing a helmet and if I don’t like wearing a helmet I won’t bike so that’s, it’s a complete vanity issue basically […].”* Alice, 50–59 years

In contrast, other participants described the busway as a viable, even better, alternative to their previous travel mode or route, using very similar criteria. Where other participants saw a *barrier* to the use of this new mode of transport, for example the discomfort of overcrowded bus travel, these participants highlighted *facilitators* related to comfort such as more generous seating or not having to negotiate traffic congestion as a driver. Commuting to work on the busway, either using the path or by bus, was described as enjoyable, relaxing, a time for reflection and less stressful than driving. This pleasant experience was discussed as encouraging initial and continued use of the busway. Previous car users Jenny, Omar and Nick all described the busway as more pleasant than driving.

*“It’s somewhere you can relax and sort of not get stressed by driving and things like that, which I find has been a real difference […].”* Nick, 50–59 years

*“If I catch it [the busway] at five to seven, I’m usually in my office by about twenty or quarter to eight. So it’s a little longer, maybe, than driving at that time of the morning, but it’s much more pleasant.”* Jenny, 60–69 years

The latter illustrates the willingness to compromise on the speed of a journey for the sake of pleasantness, thus demonstrating the importance of the value individuals place on different aspects of their journey experience.

#### A safer travel option?

The contextual and, at times, ambiguous nature of participants’ experiences, expectations and representations of the busway as a space was particularly pronounced in regards to the maintenance track. Cyclists, particularly those with less confidence or experience, appreciated the off-road nature of the maintenance track because it provoked fewer safety concerns than cycling on the roads.

*“There is just almost no chance of impact, the buses can’t get you because they’re in the busway and there are no cars allowed anywhere near.”* Liam, 50–59 years

However, the limited shelter, from trees or high banks, along the maintenance track made cycling physically demanding on occasion, and at first the new space was unlit and became very dark at night. This was described as a decisive safety concern by some.

*“We’ve spent this phenomenal amount of money on the guided busway, with this lovely facility for people to walk, cycle, run, whatever and it’s not lit and in the winter for women on their own it is intimidating. It’s intimidating at the Trumpington end and I’m not prepared to walk along it on my own.”* Helen, 50–59 years

*“I don’t mind the bad weather, but I don’t like the dark. […] it’s not a feeling of being mugged or attacked, it’s a feeling of being run over or me cycling into something.”* Zoe, 50–59 years

The resulting poor visibility, along with other issues such as occasional flooding, represented a potential cause of accidents and a barrier to use because it was experienced as intimidating and off-putting. What should have been a safe space for walking and cycling was in fact considered unsafe in particular circumstances, during later hours in winter months, and for women. These concerns were often noted by the same participants who appreciated the safety gains due to the off-road nature of the track, and for some those benefits outweighed the potential shortfalls. In part this may be explained by a gradual adaptation to and of the new environment, as the next theme will explore.

*“My friend […] had a good idea, and she actually wore a light on her bag [walking along the busway]. And I thought that was quite good, cos they’re [cyclists] coming up behind you.”* Vicky, 50–59 years

#### Adapting to and adopting environmental change

Embracing the introduction of the busway appeared to be experienced as a process involving a shift in the balance between influential factors which encouraged or discouraged behaviour change, planning and adopting the environmental change over time.

Novel aspects of the busway in particular, such as the ticketing procedure and two separate bus operators, meant that planning—especially for those new to public transport—was required.

*“You tend to have to work your journeys out, planning in advance and making sure you’ve got lots of time, buses not turning up, buses being late.”* Harry, 40–49 years

*“I have the utmost sympathy for anybody that’s not a regular bus user because it’s almost like having to be inducted into some sort of secret society because people […] worry about “Do I need the right money?” […] I mean this business about the stops in town, you pay on the bus, the stops outside town actually on the busway you have to pay at a machine before and then the machine asks you, “Which bus company do you want to travel with?” Not “Where are you going?”* Mark, 40–49 years

The process of incorporating the busway into commuting patterns appeared to be influenced by whether the anticipated benefits of changing were achieved or not over time.

*“I think you think it’s quite a long way. I know when I first started doing it [walking along the busway to the park and ride site] I thought, ‘Oh, I’ll do it once a week, twice a week,’ which is what I did, actually. […] And then I thought, ‘Well actually, I can do it every day,’ but it was a question of building up to it.”* Vicky, 50–59 years

*“I thought it’s being provided, it’s been a long time coming, I really should give it a try, and I did find that it suited me, both time, frequency, cost. […] and then it’s successful so I’ve go[ne] with it, yes.”* Jenny, 60–69 years

### Freya’s case study

The busway interacted with participants’ circumstances in a complex manner which is challenging to assimilate across many voices and lived experiences. To understand the potential of the busway to bring about changes in transport and physical activity behaviours, it is useful to highlight the experiences of one voice encapsulated in Freya’s story (Table 4), which also represents and draws together the three themes discussed above.

**Table 4:** Case study

Freya’s study
At the beginning of the study Freya, a woman in her thirties, was driving to a park-and-ride site (approximately 10 miles from her home) and then cycling approximately two miles to work (Table 3). She disliked doing this because she would spend a lot of time stationary in traffic. During the study she changed to cycling the whole journey (approximately eight miles). This change was prompted by the introduction of the busway which meant she did not have to cycle on roads for the whole journey. Taking up cycling to work also supported Freya’s motivation to lose weight and become fitter.
*“A lot of the reason why I wanted to start cycling [along the busway to work] was to lose weight and to get fit and I lost three and a half stone over two years.”*
Freya described becoming a more confident cyclist who felt more comfortable cycling on roads.
*“One of the reasons I started cycling in the first place was because the busway was built because I didn’t like the idea of cycling on the roads, now I’d say I do go along the road route quite often but I’m a much more confident cycler than I was when I first started so it doesn’t bother me as much but having that way of getting in which is mainly off-road was actually, you know, part of the reason why I felt I could cycle all the way and start doing it.”*
This feeling of confidence and competence was important because occasionally the maintenance track access path was too muddy and wet to cycle along. Therefore, Freya needed to be able to use roads to continue cycling.
*“The stretch to get from the end of my road onto the busway is just a path so it gets very muddy, especially with the wet weather that we had a few months ago, it was just completely mud and waterlogged so I couldn’t cycle along it, so when it’s very wet I go a different route which is through Oakington and Girton and then down Huntingdon Road so that’s a road route rather than an off-road route.”*

### Reflecting on behaviour: the use of vignettes

The vignettes acted as a stimulus tool which encouraged reflections on personal experiences, how those experiences related to the characters within the vignettes and hypothetical reflections on the process an individual might experience in a similar situation. For example, Hannah empathised with the vignette character’s response to environmental change.

*“Well the first one [vignette] I can see where a person would be coming from, you know. Right, the system has changed, you’re aware that it’s changed, she tried it and found it was okay […] and it needed to be a continuing good experience for the person to carry on doing it which obviously it was.”* Hannah, 50–59 years

The vignettes were a useful tool for eliciting experiences of established less consciously considered behaviours by helping participants compare their own lived experiences with that of others. This articulation furthered our understanding of the considerations implicit in travel choices.

*“I’m not that great at exercising for the sake of exercising [phrase within vignette 1] so I definitely agree with that statement that it’s a way of keeping fit because you have to, you know, you get out of that, you don’t have to sort of come home and say ‘well I’ve got to go to the gym’ […]”* Sophie, 30–39 years

This reaction also illustrates the potential of vignettes to reduce the risk of socially desirable responses. The statement about not ‘exercising for the sake of exercising’ to which this participant referred may be considered less socially desirable. However, several participants agreed with this statement, suggesting that the vignettes may have allowed participants to feel more comfortable in endorsing it.

## Discussion

This paper illustrates the diverse responses to the busway in relation to the place and space created by the new infrastructure. Although people are unlikely to use new transport infrastructure if it does not closely match the journeys they need to make (a consideration of *place*), this analysis has elicited a diverse range of salient factors informing travel behaviour which depend on the value individuals attribute to different aspects of their journey experience. As a result, in some circumstances greater weight may be given to considerations of *space*, such as perceptions of safety, above considerations related to *place* such as journey duration.

### Appeal of the places created

Experiences of using the busway depended in part on the component with which individuals interacted or their ‘geographic activity space’ [51]; for example, those travelling solely along guided sections of the busway benefitted most in terms of speed compared to those with journeys using both guideway and public roads. Thus, to understand the influence of environmental change on health behaviours it is important to identify the most salient characteristics of new infrastructure and how these features interacted with people’s activity spaces. Living nearer new or improved walking and cycling routes predicted increases in these behaviours, as well as overall physical activity, in the iConnect study [52,53]. It is plausible, therefore, that scaling up the benefits of isolated changes to transport infrastructure to the population level would require accessible, wide-spread, high quality improvements across the network of infrastructure [18,54].

### Convenience

Objective and perceived measures of convenience of routes have been found to predict the uptake of walking and cycling for commuting [47]; similarly, changes in the perceived convenience of public transport have been associated with shifting away from car commuting [55,56]. The current findings go further by suggesting a more ambiguous relationship between infrastructure provision and its use.

The track was experienced positively by some users because of its convenience and the fact that it offered a smooth off-road cycle path, in spite of the perceived safety concerns and other limitations of the maintenance track. Confirming previous findings by Guell et al [36], this study found that parking difficulties within Cambridge are likely to have encouraged the adoption of the busway for some. This is consistent with previous research which showed that people without access to car parking at work were more likely to incorporate walking and cycling into their car-commute [47,56].

Convenience, cost, comfort, speed and reliability were considered important elements in the decision-making process for those either adopting or rejecting the busway, suggesting—as others have done—that efforts to promote active commuting may be most effective when emphasising these factors rather than potential health benefits [37]. When introducing new infrastructure, significant journey characteristics such as these should be considered in relation to the competing alternatives [57]. For example, the provision of free bus travel for young people in the ‘On the Buses’ study displaced short walking and cycling trips onto public transport, suggesting a possible negative effect on active travel [58]. Therefore the physical activity impact of new transport infrastructure may depend crucially on the context within which it is introduced [24,59].

### Appeal of the spaces generated

The busway created a range of differently experienced spaces: pleasant views, leather seats, air-conditioning and internet access, or overcrowded, uncomfortable spaces; a safe, well-surfaced traffic-free route for active travel, or a poorly lit path that was susceptible to flooding. Depictions of pleasant experiences on the busway relate to previous research illustrating that journeys to work can have positive associations with wellbeing [35]. However, our findings show that the pleasantness of the busway was ambiguous and dependent on circumstances, such as the time of day, and individual preferences. For example, whilst cars offer more privacy than public transport, and overcrowded buses can be expected to present a barrier to passengers for whom personal space is important [34,57], our data confirm that some people actually valued a more sociable form of transportation [57].

### Understanding diverse responses to environmental change

The diverse responses to the busway can be attributed to the complexity of this natural experiment and the diverse contexts in which the intervention was experienced [60]. Indeed it is anticipated that interventions such as this will have multiple mechanisms (processes through which an intervention brings about outcomes) and multiple contexts in which these mechanisms operate [30]. Similarly the components of the busway were introduced in stages and continue to be modified [61], so experiences of the busway are likely to change over time.

The effect of various influential factors also appeared to be dependent on individual circumstances [30] and the value individuals attributed to certain factors. This is in line with the findings of an ethnographic exploration of the busway [38], from which Jones and colleagues inferred that it may be appropriate to target the marketing of new transport infrastructures differently to bus and car users. Similarly, Hiscock and colleagues propose that researchers need to know more about the characteristics of individuals in order to understand transport experiences [57]. The concept of shifts in the balance of influential factors when individuals are susceptible to change is supported by previous research [26] which described ‘turning points’ in cycling behaviour being ‘triggered’ by life events such as relocation of home or work or by ‘changes to the environment’. In the current study, similar mechanisms were demonstrated in a broader range of travel behaviours, suggesting that they are not unique to cycling.

### Strengths and limitations

These interviews are the first within the Commuting and Health in Cambridge study to explore longer term changes in travel behaviours after the introduction of the busway and have captured the experiences of a diverse sample, with varying levels of ‘exposure’ to the transport system, using a range of transport modes. By using qualitative methods the complex and ambiguous nature of lived travel experiences have been explored.

This research has demonstrated the utility of vignettes in interviews focusing on travel behaviour. Vignettes can raise the consciousness of travel behaviours [29,45], which are often stable [62] and somewhat habitual [63]. They may also help to move the discussion beyond an individual’s own experiences to the social context within which their behaviour occurs [44]. This is useful in the study of travel behaviour decisions which are negotiated within a social context of the behaviours of others and the travel options available [33].

In producing vignettes using data from previous research, a level of credibility and relevance was anticipated [44]. Participants often remarked how representative the vignettes were of their own views. There is a potential that some participants may have adopted the views of a vignette even if it did not reflect their own [45]. Another limitation is that a narrowly focused vignette can mean that other instances or situations are not addressed because the researcher has determined the content to be discussed [64]. For these reasons, the vignettes were employed at the end of the interview. In addition, participants sometimes contributed additional scenarios which were not covered in the vignettes presented.

The interviews nevertheless had some limitations which warrant attention. The sample included a higher proportion of cohort members than intercept survey participants, which could reflect a greater investment and commitment already made to the study. Within the main cohort, a large proportion had been educated to degree level, although the characteristics of the purposively recruited intercept participants somewhat offsets this. The interview sample did not represent the experiences of adults aged under 30, who may respond differently to particular attributes of the busway such as internet access, or have different predispositions to particular travel behaviours such as cycling. The selection of participants who had changed their travel behaviours relied on one survey item which may not have provided a valid reflection of changes in travel behaviours over time. Nevertheless, this approach was successful in eliciting a diverse range of participants amongst whom some had undergone a change in their travel behaviour.

## Conclusions

This research has examined the diverse responses to environmental change elicited by the introduction of the busway, providing some indication that environmental change supporting active travel and public transport *can* encourage behaviour change for *some* people in *certain* circumstances. It has helped us understand the characteristics of those people as well as the features of the busway which led people to either accept or reject it. Place and space features including accessibility, convenience, pleasantness and safety were important for the acceptance of the busway relative to the alternative options. While responses to the busway were diverse, they did culminate in meaningful travel behaviour change for some through a process of shifts in the balance between influential factors, planning and incorporating the environmental change into commuting practices over time.
